# Presynaptic Nicotinic α7 and Non-α7 Receptors Stimulate Endogenous GABA Release from Rat Hippocampal Synaptosomes through Two Mechanisms of Action

**DOI:** 10.1371/journal.pone.0016911

**Published:** 2011-02-08

**Authors:** Stefania Zappettini, Massimo Grilli, Federica Lagomarsino, Anna Cavallero, Ernesto Fedele, Mario Marchi

**Affiliations:** 1 Section of Pharmacology and Toxicology, Department of Experimental Medicine, University of Genoa, Genoa, Italy; 2 Center of Excellence for Biomedical Research, University of Genoa, Genoa, Italy; 3 National Institute of Neuroscience, Genoa, Italy; Institut National de la Santé et de la Recherche Médicale, France

## Abstract

**Background:**

Although converging evidence has suggested that nicotinic acetylcholine receptors (nAChR) play a role in the modulation of GABA release in rat hippocampus, the specific involvement of different nAChR subtypes at presynaptic level is still a matter of debate. In the present work we investigated, using selective α7 and α4β2 nAChR agonists, the presence of different nAChR subtypes on hippocampal GABA nerve endings to assess to what extent and through which mechanisms they stimulate endogenous GABA release.

**Methodology/Findings:**

All agonists elicited GABA overflow. Choline (Ch)-evoked GABA overflow was dependent to external Ca^2+^, but unaltered in the presence of Cd^2+^, tetrodotoxin (TTX), dihydro-β-erythroidine (DHβE) and 1-(4,4-Diphenyl-3-butenyl)-3-piperidinecarboxylic acid hydrochloride SKF 89976A. The effect of Ch was blocked by methyllycaconitine (MLA), α-bungarotoxin (α-BTX), dantrolene, thapsigargin and xestospongin C, suggesting that GABA release might be triggered by Ca^2+^ entry into synaptosomes through the α7 nAChR channel with the involvement of calcium from intracellular stores. Additionally, 5-Iodo-A-85380 dihydrochloride (5IA85380) elicited GABA overflow, which was Ca^2+^ dependent, blocked by Cd^2+^, and significantly inhibited by TTX and DHβE, but unaffected by MLA, SKF 89976A, thapsigargin and xestospongin C and dantrolene. These findings confirm the involvement of α4β2 nAChR in 5IA85380-induced GABA release that seems to occur following membrane depolarization and opening calcium channels.

**Conclusions/Significance:**

Rat hippocampal synaptosomes possess both α7 and α4β2 nAChR subtypes, which can modulate GABA release via two distinct mechanisms of action. The finding that GABA release evoked by the mixture of sub-maximal concentration of 5IA85380 plus sub-threshold concentrations of Ch was significantly larger than that elicited by the sum of the effects of the two agonists is compatible with the possibility that they coexist on the same nerve terminals. These findings would provide the basis for possible selective pharmacological strategies to treat neuronal disorders that involve the dysfunction of hippocampal cholinergic system.

## Introduction

It is well known that the activation of specific nicotinic acetylcholine receptor (nAChR) subtypes enhances the release of glutamate, noradrenaline, and acetylcholine from rodent hippocampal nerve endings [1], [2]. nAChRs seem to play a role also in the modulation of the release of GABA in the same brain area, although the specific involvement of the different nAChR subtypes may be more complex and is therefore still a matter of debate. Studies using electrophysiological techniques have demonstrated that functional α7 and α4β2 subtypes are present on terminals of rat hippocampus and trigger the release of GABA [3]–[6]. The presence of α7 nAChRs modulating GABA release has been confirmed by several authors using different experimental approaches [7]–[12]. On the contrary, some studies suggest that the β2 subunit is the component of all the nAChRs that modulate [^3^H]GABA release in mouse brain synaptosomes [13], [14], and exclude the presence of α7 nAChR subtypes. In line with these findings, Wonnacott et al. [15] reported that the nicotine-evoked [^3^H]GABA release from rat hippocampal synaptosomes was blocked by dihydro-β-erythroidine (DHβE) but not by α-bungarotoxin, suggesting that α7 nAChRs were not involved in hippocampal GABA release. However, it has to be noted that a particular α-bungarotoxin-insensitive α7 nAChR was found to mediate enhancement of GABA release from chick central nervous system [16], and that rat hippocampal α7 and β subunits can co-assemble to form functional heteromeric receptors [17], [18]. Finally, there is evidence indicating that nAChRs are present on GABAergic neurons, at least on the preterminal level [19]. Moreover, the existence of α7 and non-α7 nAChRs on nerve endings has also been challenged by the results of Kanno et al. [20], who showed that both receptor subtypes exert some modulatory effects on GABA release via a multi-synaptic control, as they do not have a sufficient potency to modulate the release under the control of a single synapse.

As for the functional diversity of nAChR subtypes, increasing evidence support the possibility that different nAChR subtypes trigger neurotransmitter release through different molecular mechanisms [21]–[23].

Using purified hippocampal synaptosomes in superfusion, in the present study we have a) investigated whether and to what extent selective α7 and α4β2 receptor agonists [5], [24]–[27] are able to evoke endogenous GABA release and b) characterised the molecular mechanisms involved in these effects. The results indicate that, in rat hippocampus nAChRs of the α7 and α4β2 subtypes are present on nerve endings and stimulates the endogenous GABA release via two distinct mechanisms of action.

## Results

**Figure 1:**
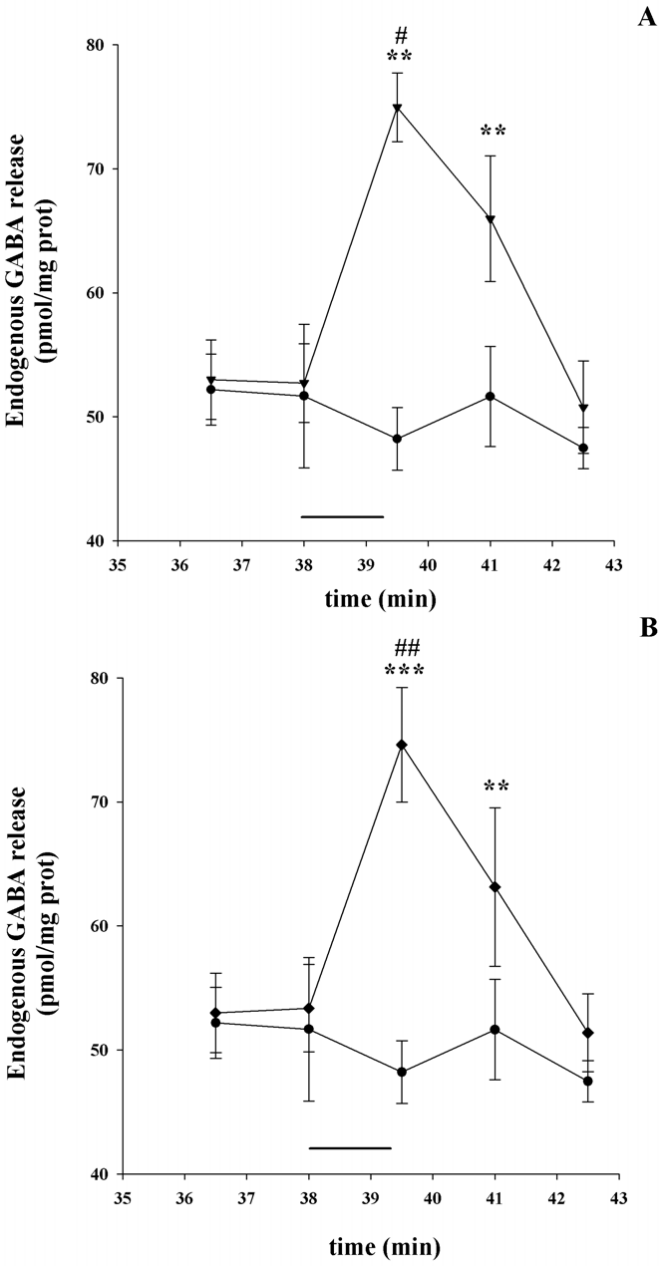
Time course of GABA release in response to different agonists. Stimulatory effects of Ch (1 mM; ▾) (A) and 5IA85380 (10 nM; ♦) (B). Values are from two experiments and represent mean ± SEM of eight replicate superfusion chambers per condition (basal or evoked release). **p<0.01, ***p<0.001 versus time 36.5; ^#^p<0.05, ^##^p<0.01 versus basal release (•). Two way ANOVA followed by Tukey-Kramer *post hoc* test.

Figure 1A,B illustrates the time course of the endogenous GABA release evoked by a 90 s pulse of choline (Ch) or 5-Iodo-A-85380 dihydrochloride (5IA85380) reported to act selectively on α7 and on α4β2 nAChR subtypes, respectively. The Ch- and the 5IA85380-evoked release of GABA showed a similar pattern reaching a maximum corresponding to min 39.5 of superfusion and decline to basal level at min 42.5 (Fig 1A,B). Since a certain degree of desensitization may occur through the stimulation period of 90 s it is possible that we are underestimating the stimulatory effect of the two agonists on the GABA release.

Table 1 shows the effects of four different nicotinic agonists on endogenous GABA release from rat purified hippocampal synaptosomes in superfusion. In this study, we have used two α7 selective agonists Ch and PHA543613 hydrochloride (PHA543613), and the α4β2 selective compounds 5IA85380 and RJR2429 dihydrochloride (RJR2429). The GABA overflows elicited by Ch (1 mM) and PHA543613 (100 µM) were respectively 41.25±2.76 and 32.54±2.08 and closely resemble those elicited by the two selective α4β2 receptor agonists 5IA85380 (10 nM) and RJR2429 (3 µM) (40.43±3.71 and 32.35±5.67 respectively). The stimulatory effects of these four agonists have been compared to the GABA overflow evoked by depolarization with 9 and 15 mM KCl. In the presence of 9 and 15 mM KCl in the perfusion solution, the GABA overflows were 47.22±5.98 and 109.13±4.02, respectively. Therefore, the amount of endogenous GABA released by all the four nicotinic agonists was quantitatively very similar to that released by the lower concentration of KCl (9 mM).

**Table 1:** Effects of selective nAChR subtype agonists on endogenous GABA overflow from rat hippocampal synaptosomes.

Drugs	Endogenous GABA overflow (pmol/mg prot)
α7 *nAChR subtype agonists*	
Ch (1 mM)	41.25±2.76
PHA543613 (100 µM)	32.54±2.08
*α4*β*2 nAChR subtype agonists*	
5IA85380 (10 nM)	40.43±3.71
RJR2429 (3 µM)	32.35±5.67
KCl (9 mM)	47.22±12.98
KCl (15 mM)	109.13±4.02##

Data are means ± SEM of three experiments run in triplicate. For experimental details see Materials and Methods.

## p<0.01 versus KCl (9 mM).

One way ANOVA followed by Tukey-Kramer *post hoc* test.

**Figure 2:**
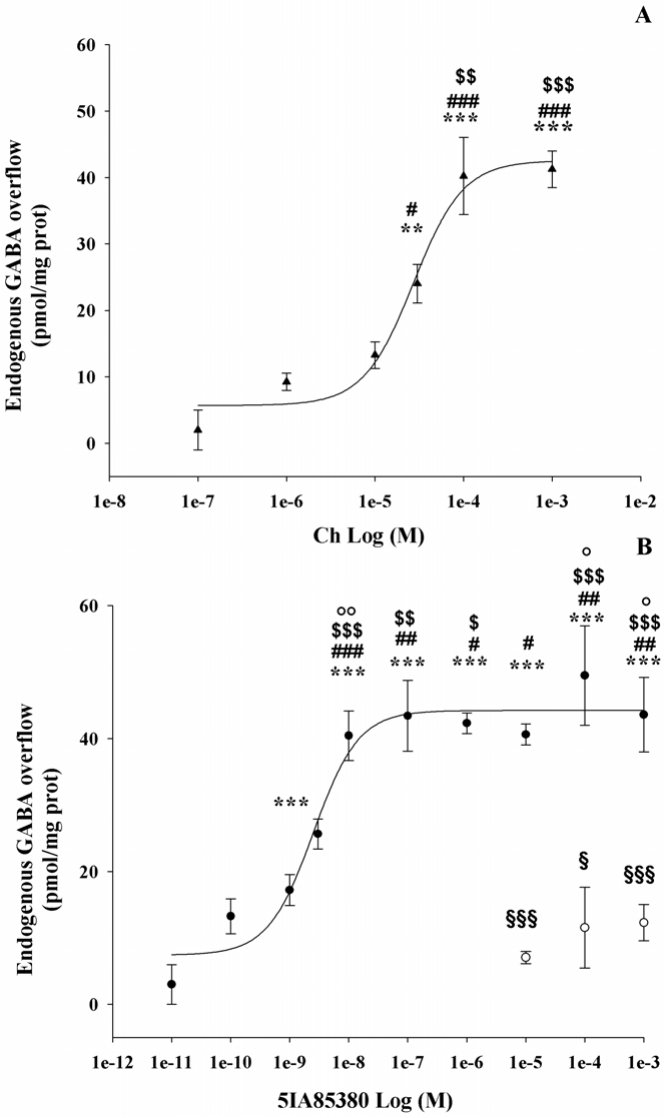
Concentration-dependent effect of Ch and 5IA85380 on endogenous GABA overflow from rat hippocampal synaptosomes. (**A**) Data are mean ± SEM of 3–6 experiments for each concentration run in triplicate. **p<0.01, ***p<0.001 versus Ch (100 nM); ^#^p<0.05, ^###^p<0.001, versus Ch (1 µM); ^$$^p<0.01, ^$$$^p<0.001 versus Ch (10 µM). One way ANOVA followed by Tukey-Kramer *post hoc* test (Ch  = ▴). (**B**) Data are mean ± SEM of 3–6 experiments for each concentration run in triplicate (three superfusion chambers for each experimental condition). ***p<0.001 versus 5IA85380 (10 pM); ^#^p<0.05, ^##^p<0.01 ^###^p<0.001 versus 5IA85380 (100 pM); ^$^p<0.05, ^$$^p<0.01, ^$$$^p<0.001 versus 5IA85380 (1 nM); °p<0.05, °°p<0.01 versus 5IA85380 (3 nM); ^§^ p<0.05, ^§§§^ p<0.001 versus 5IA85380 alone respectively. One way ANOVA followed by Tukey-Kramer *post hoc* test (5IA85380  =  •; 5IA85380 + DHβE 2 µM  =  ○)

When synaptosomes were exposed to various concentrations of Ch (0.01 mM–1 mM) or 5IA85380 (0.1 nM–1 mM), both nicotinic agonists were found to increase GABA overflow in a concentration-dependent manner, the apparent EC_50_ values for Ch and 5IA85380 being 12.06±0.66 µM (Hill coefficient: 1.5) and to 2.51±0.91 nM (Hill coefficient: 1.14), respectively (Fig. 2A,B). In order to investigate on the presence of low affinity α4β2 nAChR subtypes we extended the concentration-response curve of 5IA85380 in presence and in absence of 2 µM DHβE. The result show that 5IA85380 at higher concentrations (from 1 µM to 1 mM) produced a stimulatory effect of endogenous GABA release similar to that produced at 10 nM. This effect was completely blocked in presence of 2 µM DHβE (Fig. 2B).

**Figure 3:**
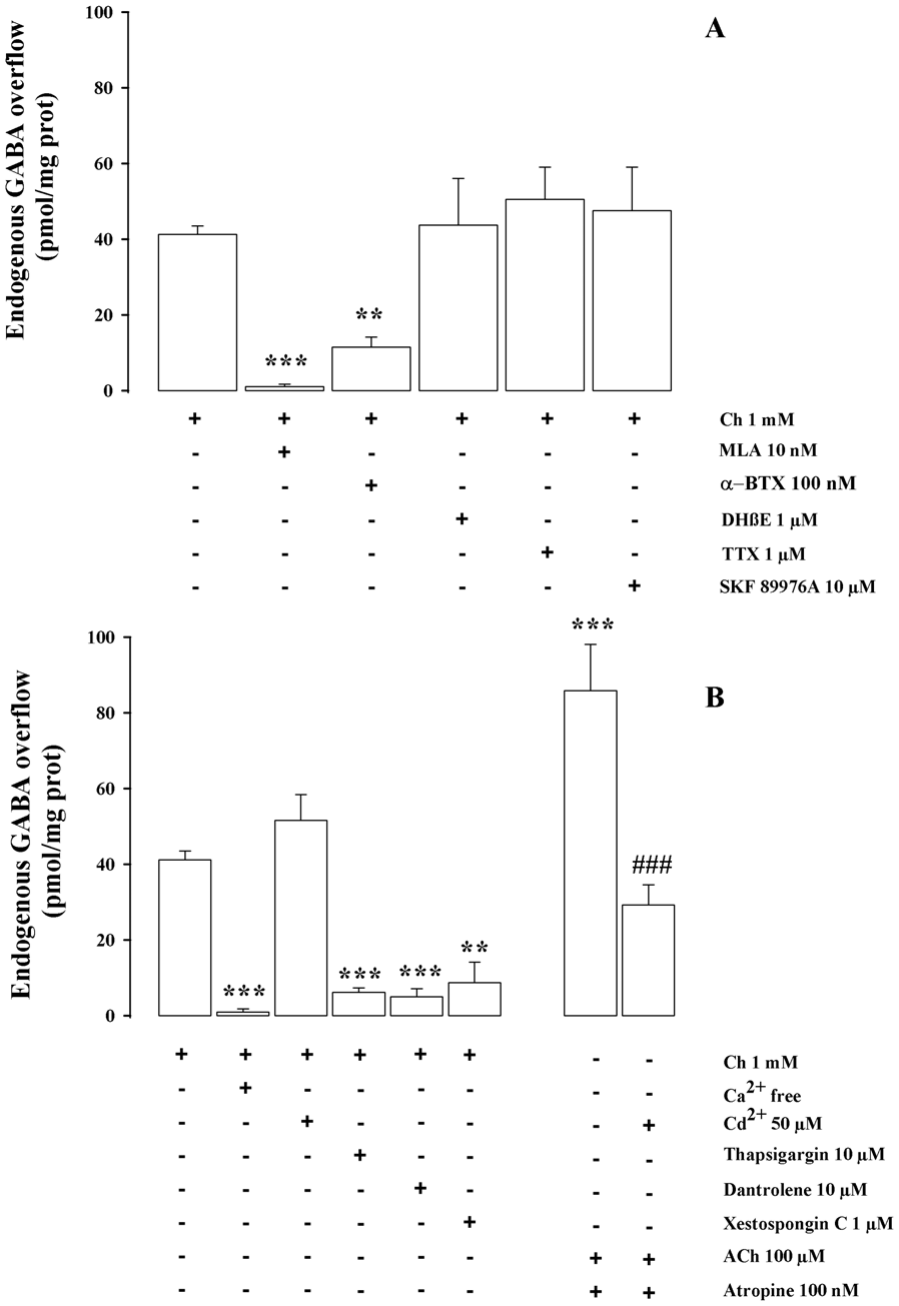
Characterization of Ch-evoked endogenous GABA release. (**A**) Effect of MLA, (α-BTX, DHβE, TTX and SKF 89976A on endogenous GABA overflow evoked by Ch from rat hippocampal synaptosomes. Synaptosomes were depolarised with Ch for 90 s at *t* = 38 min of superfusion. When appropriate antagonists were introduced 8 min before depolarization. Data are mean ± SEM of 3–6 experiments run in triplicate. ***p<0.001, **p<0.01 versus Ch-evoked GABA overflow. One way ANOVA followed by Dunnett *post hoc* test. (**B**) Effect of Ca^2+^ free, Cd^2+^, thapsigargin, dantrolene, and xestospongin C on endogenous GABA overflow evoked by Ch (1 mM) or by ACh plus atropine from rat hippocampal synaptosomes. When appropriate, Ca^2+^ was omitted 18 min before Ch. Data are mean ± SEM of 3–6 experiments run in triplicate. **p<0.01, ***p<0.001 versus Ch-evoked GABA overflow,^ ###^p<0.001 versus ACh evoked GABA overflow. One way ANOVA followed by Dunnett *post hoc* test.

The Ch (1 mM)-evoked release was significantly antagonised by methyllycaconitine (MLA; 10 nM), α-bungarotoxin (α-BTX, 100 nM) and unaffected by DHβE (1 µM) confirming the involvement of an α7 nAChR. The voltage operated Na^+^ blocker tetrodotoxin (TTX) (1 µM) and SKF 89976A (10 µM), a specific inhibitor of the GABA carrier, did not modify the Ch (1 mM)-evoked GABA overflow (Fig. 3A). The ability of Ch (1 mM) to evoke GABA overflow was totally external Ca^2+^-dependent but was insensitive to the non specific voltage-operated Ca^2+^ channel (VOCC) blocker Cd^2+^ (50 µM), suggesting that Ca^2+^ entry into synaptosomes following Ch exposure might occur through the α7 nAChR channel. Conversely, the effect of choline (1 mM) was abolished by dantrolene (10 µM), thapsigargin (10 µM) and xestospongin C (1 µM) a finding consistent with a fundamental role of intracellular calcium stores in mediating the α7 response (Fig. 3B). The natural neurotransmitter ACh (100 µM), tested in presence of atropine (0.1 µM) to avoid muscarinic effects, produced an increase of the GABA release quantitatively similar to the sum of the effects produced by Ch and 5IA85380 and its stimulatory effect was partially inhibited (−60%) by CdCl_2_ (50 µM).

**Figure 4:**
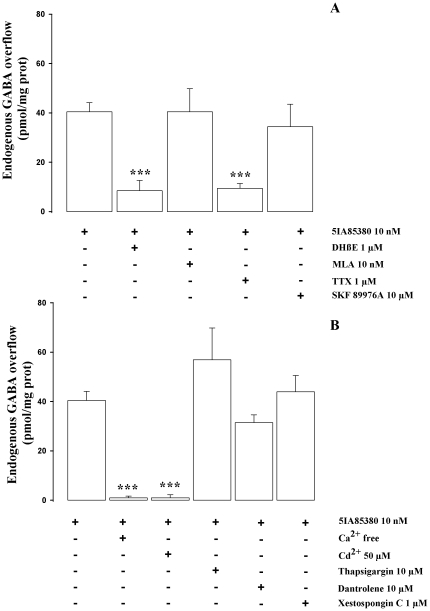
Characterization of 5IA85380 endogenous GABA release. (**A**) Effect of DHβE, MLA, TTX and SKF 89976A on endogenous GABA overflow evoked by 5IA85380 from rat hippocampal synaptosomes. Synaptosomes were depolarised with 5IA85380 for 90 s at *t* = 38 min of superfusion. When appropriate, antagonists were introduced 8 min before depolarization. Data are mean ± SEM of 3–6 experiments run in triplicate. ***p<0.001 versus 5IA85380 evoked GABA overflow. One way ANOVA followed by Dunnett *post hoc* test. (**B**) Effect of Ca^2+^ free, Cd^2+^ and thapsigargin, dantrolene, and xestospongin C on endogenous GABA overflow evoked by 5IA85380 from rat hippocampal synaptosomes. When appropriate, antagonists were introduced 8 min before 5IA85380. Data are mean ± SEM of 3–6 experiments run in triplicate. ***p<0.001 versus 5IA85380 evoked GABA overflow. One way ANOVA followed by Dunnett *post hoc* test.

The 5IA85380 (10 nM)-evoked GABA overflow was blocked by DHβE (1 µM) (−79%) and unaffected by MLA (10 nM), confirming the involvement of an α4β2 nAChR subtype. The effect of 5IA85380 (10 nM) was significantly inhibited (−74%) in presence of TTX (1 µM) but unaffected by the specific inhibitor of the GABA carrier SKF 89976A (10 µM) (Fig. 4A). The effect of 5IA85380 (10 nM) was almost totally Ca^2+^ dependent, blocked by Cd^2+^ (50 µM) while no significant attenuation of the stimulatory effect of 5IA85380 (10 nM) was found in presence of dantrolene (10 µM), thapsigargin (10 µM) and xestospongin C (1 µM), thus excluding a significant involvement of calcium release from intracellular calcium stores following activation of the α4β2 nAChR (Fig. 4B). DHβE (1 µM), MLA (10 nM), TTX (1 µM), CdCl_2_ (50 µM) and dantrolene (10 µM) thapsigargin (10 µM) and xestospongin C (1 µM) did not produce any significant effect on basal GABA release on their own.

**Figure 5:**
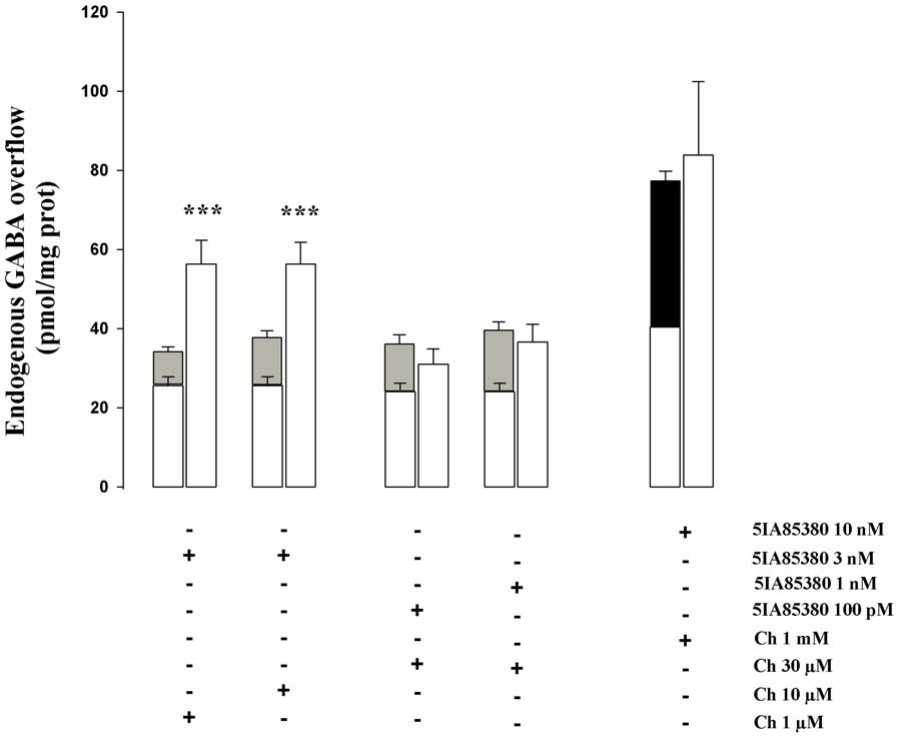
Effect of the simultaneous presence of 5IA85380 and Ch in the superfusion fluid on endogenous GABA overflow from rat hippocampal synaptosomes. Synaptosomes were stimulated simultaneously with 5IA85380 plus Ch at different concentrations for 90 s at *t* = 38 min of superfusion. Data are mean ± SEM of 4–6 experiments run in triplicate. In order to evaluate whether the effect of the co-administration of the two agonists was significantly different compared to the sum of the two agonists alone, we have added, in each experiment, to the stimulatory effect of 5IA85380 (3 nM) the average effect of subthreshold concentration of Ch alone (gray column) and, similarly to the effect of Ch (30 µM) the effect of submaximal concentrations of 5IA85380. ***p<0.001 versus their respective controls (the sum of 5IA85380 plus Ch). We have also tested the stimulatory effect of 5IA85380 plus Ch (black bar) at their maximal concentration. Two-tailed Student's *t*-test.

The simultaneous presence of 5IA85380 (3 nM) in the superfusion fluid, with two subthreshold concentrations of Ch (1 µM and 10 µM), provoked a synergistic GABA overflow that was significantly larger than that elicited by the sum of the effects of the two agonists alone (Fig. 5). The co-administration of maximal concentration of 5IA85380 (10 nM) and Ch (1 mM) produced only an additive effect. The presence of Ch (30 µM) plus two subthreshold concentration of 5IA85380 (1 nM and 0.1 nM) did not show any synergistic or antagonistic effects (Fig. 5).

**Figure 6:**
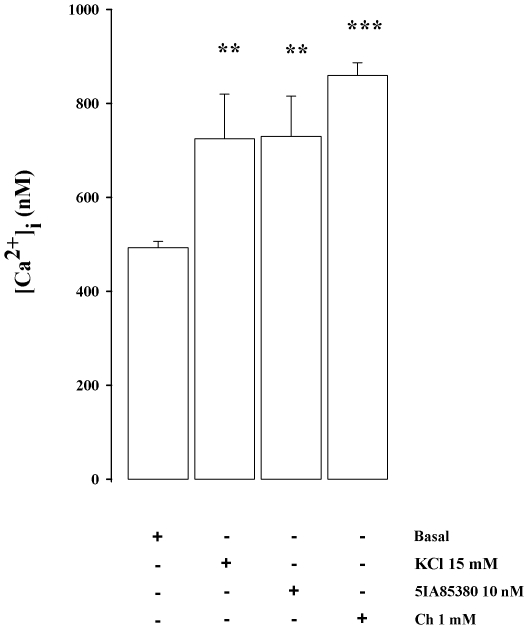
Effect of 15 mM KCl, Ch and 5IA85380 on the cytosolic Ca^2**+**^ concentration [Ca^2**+**^]i induced in rat hippocampal synaptosomes. Synaptosomes were loaded with FURA PE-3 AM, resuspended in standard HEPES-buffered medium and incubated for 40 min before fluorometric measurements. Basal Ca^2+^ levels were measured for 2 min before addition of Ch, 5IA85380 or KCl. Data are expressed as means ± SEM of three experiments run in duplicate. ** p<0.01, *** p<0.001 vs. basal [Ca^2+^]i in synaptosomes (One way ANOVA followed by Dunnett Multiple Comparison Test).

Finally, as illustrated in Fig. 6, both 5IA85380 (10 nM) and Ch (1 mM) increased significantly the apparent [Ca^2+^]i in synaptosomes. The increase produced by the two agonists was similar compared to that produced by 15 mM KCl.

## Discussion

In the present work, we investigated the effect of nAChR activation on the release of endogenous GABA from rat purified isolated hippocampal synaptosomes. We have shown that selective α4β2 and α7 receptor agonists produced enhancement of endogenous GABA release through two distinct intracellular mechanisms.

Our results obtained with the specific α7 agonist Ch show that this drug was able to elicit endogenous GABA release from purified hippocampal synaptosomes at concentrations (EC_50_  = 12.06±0.66 µM) quite different from those (EC_50_  = 1.6 mM) necessary to activate the α7 nAChRs in cultured hippocampal neurons [4], [5]. This stimulatory effect was antagonised by MLA, α-BTX and not by DHβE indicating that, although obtained at very low concentration, the release seems to be mediated by a α7 nAChR subtype. This Ch-mediated GABA release was not inhibited in the presence of TTX, therefore excluding a major involvement of the voltage dependent sodium channels. On the other hand, the TTX-insensitive carrier-independent enhancement of GABA release by Ch was both dependent on external calcium and prevented by thapsigargin, xestospongin C and dantrolene. Therefore, the following possible sequence of events underlying the TTX-insensitive, choline-induced GABA release can be suggested. First Ch, even at very low concentration, can increase Ca^2+^ influx directly through the α7 nAChR channel, which is known to exhibit a high permeability for Ca^2+^ [7], [28]. The influx of this cation may initiate a Ca^2+^-induced Ca^2+^ release from the endoplasmic reticulum stores that, finally, generates the increase of GABA release. The data showing the effects of Ch on cytoplasmic Ca^2+^ concentration (Fig. 6) support this sequence of events. Moreover in line with our results, it has been reported that, in the absence of incoming potentials, Ca^2+^ influx into hippocampal mossy fibers through α7 nAChRs triggers Ca^2+^-induced Ca^2+^ release from presynaptic stores, which induces a marked increase of glutamate release leading to high frequency bursts of mEPSCs in CA3 pyramidal neurons [29].

However, since α7 nAChRs are also permeable to Na^+^, it might be argued that their influx through the receptor channel could cause a TTX-insensitive depolarization followed by VOCC opening, entry of extracellular calcium, subsequent Ca^2+^-induced Ca^2+^ release from intracellular stores, and stimulation of GABA release. Such chain of reactions, however, seems unlikely to occur, as the broad spectrum blocker of VOCCs Cd^2+^ was not able to affect the Ch-induced release of GABA.

Similar results were recently obtained in pre-fontal cortex synaptosomes and hippocampal mossy fiber terminals, where activation of α7 nAChRs by nicotine has been shown to enhance glutamate release in an extracellular Ca^2+^-dependent and VOCCs-independent manner and without causing membrane depolarization [22], [23]. Also in their studies, α7 nAChRs-mediated Ca^2+^-induced Ca^2+^ release seems to represent the key mechanism capable of inducing glutamate exocytosis.

As far as the low concentrations of Ch able to elicit GABA release, it should be noted that this agonist elicits different nicotinic responses according to the concentration used [5]. Indeed, at concentrations between 50–300 µM, Ch was found to cause mild activation of α7 nAChRs and an increase of Ca^2+^ influx in neurons able to induce a cascade of intracellular metabotropic functions; however, at these low concentrations, the activation of α7 nAChRs was not sufficient to induce excitation of hippocampal interneurons [6]. Our observation that the α7 nAChRs are sensitive to low concentration of Ch could be explained by the fact that access of Ch to receptors is of course easier in synaptosomes than in slices. Indeed, it has been shown, in electrophysiological experiments on hippocampal GABAergic interneurons that Ch induces action potentials by activating putative α7 nAChRs either at high (mM) concentration after short (tens of millis) exposure or at low (µM) concentration with long (10–20 sec) exposure [5]. It is therefore likely that the apparent EC_50_ of Ch or for α7 nAChRs is lower in synaptosomes than in electrophysiological preparations in view of the longer exposure time and higher efficacy of the agonist in the former preparation. However the possibility that at least in part some of these receptors, which are not completely blocked by α-BTX (Fig. 3), are different from the typical α7 nAChRs [5], [16] has to be also taken in consideration.

Beside α7 nAChRs, our results demonstrate that hippocampal GABAergic nerve terminals also possess functional α4β2 nAChRs. In fact, in our experiments, the selective α4β2 receptor agonist 5IA85380 was able to elicit endogenous GABA release from purified hippocampal synaptosomes with an EC_50_ in the range of the high affinity for nAChRs as previously reported [30]. It is interesting to note that a low affinity nAChRs, DHβE insensitive, which modulate rubidium efflux have been demonstrated to be present in rat hippocampus [30]. As reported these receptors represent a new and previously undescribed nAChRs which may be of significant [30].

Our results using high concentrations of 5IA85380 show that the stimulatory effects on GABA release of this compound was similar to that produced by lower concentrations (10 nM) and was always inhibited in presence of 2 µM DHβE. In our experimental conditions it seems therefore unlikely that the DHβE insensitive, α4β2 nAChRs are significantly involved in the stimulation of endogenous GABA release from hippocampal isolated nerve endings. However, the possibility that these receptors, previously demonstrated monitoring Rubidium efflux from rat hippocampal synaptosomes, could be present on other selective neuronal population has to be also considered.

As for the molecular mechanisms, the 5IA85380-evoked, carrier-independent endogenous GABA release was external Ca^2+^-dependent and completely blocked by Cd^2+^, while dantrolene, thapsigargin and xestospongin C were devoid of any effect, indicating that opening of VOCCs, but not Ca^2+^ release from intracellular stores, is an obligatory step in the α4β2-evoked facilitation of GABA release.

Interestingly, the 5IA85380-evoked GABA overflow was abolished in the presence of TTX, demonstrating that opening of voltage dependent sodium channels is required for the α4β2 nAChR effect to occur. At a first glance, this result seems at odds with the general notion that presynaptic receptor-mediated effects are TTX-insensitive and since TTX has not been shown to be active at any nAChR subtype tested [31]. However, this view has been challenged by different studies showing that presynaptic nAChR effects are external sodium-dependent and can be largely prevented by the selective voltage dependent sodium channel toxin [32]–[34]. Therefore, it is generally accepted that neurotransmitter release evoked by the activation of non-α7 nAChRs is a Na^+^- and Ca^2+^-dependent process and is mediated by N- and/or P/Q-type of VOCCs [22], [35], [36] with a lack of L-type VOCCs involvement [22].

The present study, therefore, demonstrates that, in the rat hippocampus, two nAChR subtypes are present on GABAergic nerve endings where they induce enhancement of endogenous GABA release via two distinct mechanisms. While the physiological significance of two different nAChRs both modulating hippocampal basal release of GABA could be difficult to explain their role in the mechanism of action of nicotine could be relevant.

The hippocampus contains many sources of GABA nerve terminals, including those derived both from GABAergic interneurons and from septo-hippocampal GABAergic afferents. Previous results have demonstrated that 65% of GABA hippocampal interneurons express α7 receptors while only 35% express the α4β2 subtypes [3] and that nAChR activation excites distinct subtypes of hippocampal interneurons [37], [38].

Nevertheless, the possibility that at least a certain amount of synaptosomes (although a minority), derived from the septo-hippocampal GABAergic projections, possesses nAChR subtypes can not be ruled out. Indeed, nAChRs have been found in a proportion of GABAergic cells innervating hippocampal interneurons and recent data of *in situ* hybridization study showed the presence of α7 and β2 nAChR subunit mRNAs in most GABAergic neurons in the medial septum [39]–[41]. Although these receptors have been identified mostly on somatic and dendrites membrane components of the medial septal neurons, the possibility that they are present also on the nerve endings is very likely. A recent approach using *in situ* hybridization corroborates our neurochemical findings, in particular confirms the presence of specific receptor subtypes on GABAergic neurons [42].

In order to demonstrate the possible co-expression and co-operation of α4β2 and α7 nAChR subtypes on the same nerve endings we have performed some experiments varying the concentrations of the two agonists together using subthreshold concentration of one agonist in presence of submaximal concentration of the other and viceversa. Our results show that the α4β2 nAChRs may exert a permissive role on the activation of Ch mediated GABA release (Fig. 5) suggesting that, at least in part, the α4β2 and α7 nAChRs may coexist on the same nerve endings. This synergistic effect did not occur when GABA release was stimulated by submaximal concentration of Ch in presence of subthreshold concentrations of 5IA85380. This finding favor the idea that the TTX-sensitive depolarization of the nerve endings elicited by the activation of the α4β2 nAChRs may play an important role in the synergistic interaction as previously reported [43], [44] for a review see also [45].

Although both α4β2 and α7 nAChRs are present on nerve endings and elicit GABA release, it can be also hypothesised that they are differently located on the membranes of the nerve terminals and subserve different presynaptic functional roles. The fact that α7 nAChRs respond to low concentrations of Ch open up the possibility that they might be activated by volume transmission in a non synaptic manner by the diffusing Ch, which derives from acetylcholine hydrolysis [46], [47].

The understanding of the physiological role of these nAChRs and the definition of their location would provide the basis for possible selective pharmacological strategies to treat neuronal disorders, which involve the disruption of the normal function of the hippocampal cholinergic system.

## Materials and Methods

### Animals and brain tissue preparation

Adult male Sprague–Dawley rats (200–250 g) were housed at constant temperature (22 ± 1°C) and relative humidity (50%) under a regular light–dark schedule (light 7 a.m.–7 p.m.). Food and water were freely available. The animals were killed by decapitation and the hippocampus rapidly removed at 0–4°C. The experimental procedures were approved by the Ethical Committee of the Pharmacology and Toxicology Section, Department of Experimental Medicine, in accordance with the European legislation (European Communities Council Directive of 24 November 1986, 86/609/EEC) and were approved by Italian legislation on animal experimentation (Decreto Ministeriale number 124/2003-A). All efforts were made to minimize animal suffering and to use the minimal number of animals necessary to produce reliable results.

### Experiments of release

Purified synaptosomes were prepared on Percoll® gradients (Sigma-Aldrich, St Louis, MO, USA) essentially according to Nakamura et al. [48], with only minor modifications. Briefly, the tissue was homogenised in 6 volumes of 0.32 M sucrose, buffered at pH 7.4 with Tris–HCl, using a glass-teflon tissue grinder (clearance 0.25 mm, 12 up–down strokes in about 1 min). The homogenate was centrifuged (5 min, 1000 *g* at 4°C) to remove nuclei and debris; the supernatant was gently stratified on a discontinuous Percoll® gradient (2%, 6%, 10%, and 20% v/v in Tris-buffered sucrose) and centrifuged at 33500 *g* for 5 min at 4°C. The layer between 10% and 20% Percoll® (synaptosomal fraction) was collected, washed by centrifugation and resuspended in physiological HEPES-buffered medium having the following composition (mM): NaCl 128, KCl 2.4, CaCl_2_ 3.2, KH_2_PO_4_ 1.2, MgSO_4_ 1.2, HEPES 25, pH 7.5, glucose 10, pH 7.2–7.4 [13]. Synaptosomal protein content following purification was 10–15% of that in the supernatant stratified on the Percoll® gradient.

The synaptosomal suspension was layered on microporous filters at the bottom of a set of parallel superfusion chambers maintained at 37°C ([49]; Superfusion System, Ugo Basile, Comerio, Varese, Italy). Synaptosomes were superfused at 1 ml/min with standard physiological medium as previously described. The system was first equilibrated during 36.5 min of superfusion; subsequently, four consecutive 90 s fractions of superfusate were collected. Synaptosomes were exposed to agonists for 90 s starting from the second fraction collected (*t* = 38 min), with antagonists being added 8 min before agonists. The evoked overflow was calculated by subtracting the corresponding basal release from each fraction and was expressed as pmol/mg of synaptosomal proteins. We have previously amply demonstrated that in our superfusion system the possible effects of drugs operated indirectly by other mediators in the monolayer of synaptosomes in superfusion are absolutely minimised [46].

### Endogenous GABA determination

Endogenous GABA was measured by high performance liquid chromatography analysis following precolumn derivatization with *o*-phthalaldehyde and resolution through a C18-reverse phase chromatographic column (10×4.6 mm, 3 µm; Chrompack, Middleburg, The Netherlands) coupled with fluorometric detection (excitation wavelength 350 nm; emission wavelength 450 nm). Homoserine was used as internal standard. Buffers and gradient program were prepared and executed as follows: solvent A, 0.1 M sodium acetate (pH 5.8)/methanol, 80∶20; solvent B, 0.1 M sodium acetate (pH 5.8)/methanol, 20∶80; solvent C, sodium acetate (pH 6.0)/methanol, 80∶20; gradient program, 100% C for 4 min from the initiation of the program; 90% A and 10% B in 1 min; 42% A and 58% B in 14 min; 100% B in 1 min; isocratic flow 2 min; 100% C in 3 min; flow rate 0.9 ml/min.

### Monitoring cytoplasmic Ca^2+^ concentration

Cytoplasmic Ca^2+^ concentration ([Ca^2+^]_i_) was monitored in purified synaptosomes using the fluorescent dye FURA PE 3-AM [50]. Synaptosomes were incubated for 40 min at 37°C in the dark, while gently shaking, in a medium containing 20 mM of CaCl_2_ and 5 mM FURA PE 3-AM (and 1% DMSO). Control synaptosomes containing 1% DMSO, but no FURA PE 3-AM, were prepared to measure auto-fluorescence. Synaptosomal suspension was washed to remove extra-particle FURA PE 3-AM. Pellets were resuspended in ice-cold medium, divided into 200 µl aliquots (each containing 200 mg protein) and stored on ice until use. Measures were obtained within 2 h. To estimate the apparent ([Ca^2+^]_i_), a 200 µl aliquot of synaptosomes was diluted into 1.8 ml of physiological medium, containing 3.2 mM CaCl_2_, and incubated at 37°C for 5 min. Fluorescence was recorded for at least 1 min before addition of 15 mM KCl or 10 nM 5IA85380 or 1 mM Ch. Measurements were made at 37°C in a thermostated cuvette under continuous stirring using a RF-5301PC dual wavelength spectrofluorometer (Shimadzu, Japan) and by alternating the excitation wavelength of 340 and 380 nm. Fluorescent emission was monitored at 510 nm. Calibration of the fluorescent signals was performed at the end of each experiments by adding 10 mM ionomycin in the presence of 3.2 mM Ca^2+^, to obtain Fmax, followed by 10 mM EGTA (adjusted to pH 8 with 3 mM Tris), to obtain Fmin [50]. Intrasynaptosomal FURA PE 3-AM was determined for each synaptosomal preparation by adding 40 mM Mn^2+^ to quench the extracellular fluorescence; this Mn^2+-^quenched fluorescence comprised 7–10% of the total fluorescence at two wavelengths and was stable for the duration of the experiments. After correcting for the extracellular dye, [Ca^2+^]_i_ was calculated by the equation of Grynkiewicz et al. [50], using a KD of 250 nM for the Ca^2+^/FURA PE 3-AM complex.

### Statistical analysis

Multiple comparisons were performed with one-or two way ANOVA followed by an appropriate *post hoc* test (Dunnett and Tukey-Kramer). Direct comparison between two groups were performed with two-tailed Student's *t*-test. Data were considered significant for p<0.05, at least. The EC_50_ and Hillslope have been calculated according to a four parameter logistic curve equation [y =  min+max–min/1+(x/EC_50_)^Hillslope^] of Sigma Plot 8.0 (Jandel Scientific, San Rafael, CA, USA).

### Chemicals

Percoll®, Choline Iodide, CdCl_2_, dantrolene, dimethyl sulfoxide, FURA PE 3-AM and α-bungarotoxin (Sigma-Aldrich, St Louis, MO, USA); 5-Iodo-A-85380, RJR2429 dihydrochloride, PHA543613 hydrochloride, dihydro-β-erythroidine hydrobromide, methyllycaconitine citrate, thapsigargin and SKF 89976A hydrochloride (Tocris Bioscience, Bristol, UK); TTX (Ascent Scientific, Princeton, NJ, USA); xestospongin C (Inalco, Milan, Italy).
